# Public disclosures of mental health problems on social media and audiences’ self-reported anti-stigma effects

**DOI:** 10.1093/heapro/daae204

**Published:** 2025-01-21

**Authors:** Zhongjie Zhang, Nicola Reavley, Gregory Armstrong, Amy Morgan

**Affiliations:** Center for Mental Health and Community Wellbeing, Melbourne School of Population and Global Health, The University of Melbourne, 207 Bouverie Street, Carlton, Victoria 3053, Australia; Center for Mental Health and Community Wellbeing, Melbourne School of Population and Global Health, The University of Melbourne, 207 Bouverie Street, Carlton, Victoria 3053, Australia; Nossal Institute for Global Health, Melbourne School of Population and Global Health, The University of Melbourne, 32 Lincoln Square, Carlton, Victoria 3053, Australia; Center for Mental Health and Community Wellbeing, Melbourne School of Population and Global Health, The University of Melbourne, 207 Bouverie Street, Carlton, Victoria 3053, Australia

**Keywords:** mental illness, social media, disclosure, narratives, prejudice

## Abstract

Public disclosures of mental health problems on social media represent a potentially powerful informal avenue for increasing mental health literacy and reducing public stigma in relation to people with mental health problems. We investigated whether the audience reported any reduction in their own stigma toward people with mental health problems after exposure to disclosures. We also examined whether self-reported stigma reduction was associated with the characteristics of audience members, the disclosers and the disclosure messages. We used Prolific to obtain a convenience sample (*N* = 803) of adults who had been exposed to a disclosure. We administered an online survey to participants and conducted a series of logistic regressions to identify any associations between disclosure-related characteristics and audience self-reported stigma reduction. Our findings showed that certain aspects of the messaging process appeared to be associated with stigma reduction. These included explicit diagnoses from disclosers, particular message themes such as psychosocial causes of mental ill health, and positive and echoing comments from other users. In addition, audience members who reported greater levels of empathy toward, perceived similarity to and identification with disclosers tended to report reduced stigma. These findings contribute to the evidence base underpinning how, when and which public disclosures of mental health problems on social media have the potential for stigma reduction. They can further help inform future health promotion practices on social media aiming to mitigate mental health-related stigma at the population level. Future research may focus more on the dynamics and match between disclosers and audiences and their effects on stigma.

**Contribution to Health Promotion**

- Public disclosures of mental health problems on social media present an opportunity for anti-stigma health promotion efforts.
- Explicit diagnoses, particular themes (e.g. psychosocial causes of mental ill health) and supportive peer comments in disclosure messages are associated with audience self-reported stigma reduction.
- Considerations of dynamics and trait match between the disclosers and the audiences are necessary when designing mental health promotion messages.
- Digital practices such as health campaigns on social media in under-resourced areas seem to be promising.

## BACKGROUND

Public stigma in relation to people with mental health problems is characterized by stereotyped beliefs (e.g. beliefs of dangerousness toward people with mental health problems), emotional responses (e.g. anxiety and fear) and discriminatory behaviors (e.g. discrimination and avoidance) (Thornicroft et al., 2007). It profoundly restricts the life opportunities of people with mental health problems including in domains such as employment, healthcare and education (Thornicroft et al., 2022). In response, various interventions have been designed and implemented to combat mental health-related stigma (Thornicroft *et al*., 2022). Anti-stigma public health campaigns, a typical example of health promotion practices, are common and often involve disclosures of mental health problems from individuals with lived experience of mental ill health. Moreover, when an individual publicly discloses their health conditions, the disclosure itself may end up as a de facto public health campaign, particularly when the person has a large following such as a celebrity (Myrick *et al*., 2024).

In the digital age, social media has empowered individuals to publicly share personal feelings and experiences from their own perspectives. Public disclosures of mental health problems contribute to these online narratives, especially as some individuals with mental health problems prefer to express their feelings or seek help via social media rather than through face-to-face communication (Naslund *et al*., 2016; Bucci *et al*., 2019). In addition, the pervasiveness of social media in our daily life enhances the visibility and outreach of these disclosures, whether they come from a ‘traditional’ celebrity—a widely recognized movie star or athlete (e.g. the famous rapper Kid Cudi disclosing his depression on Facebook), an ‘emerging’ influencer—often a grassroots social media user who has gained a large following due to their content or lifestyles (Lee *et al*., 2021) (e.g. the YouTuber Zoella disclosing her anxiety in various social media posts), or an ‘ordinary’ individual—a peer in real life (Gronholm and Thornicroft, 2022). According to intergroup contact theory (Allport, 1954; Pettigrew and Tropp, 2006), exposure to online disclosures of mental health problems creates opportunities for outgroup members to indirectly interact with ingroup members (Makhmud *et al*., 2022). This process may increase familiarity and empathy toward people with mental health problems, reduce stereotypical beliefs among outgroup members and contribute to stigma reduction (Clement *et al*., 2013; Corrigan and Nieweglowski, 2019; McCullock and Scrivano, 2023). As a consequence, strategically utilizing public disclosures of mental health problems on social media as part of campaign efforts presents an opportunity for health promotion to alleviate stigmatizing attitudes and behavior toward people with mental health problems (Betton *et al*., 2015; Zhang *et al*., 2024).

Public health campaigns essentially communicate health-related information. From the transmission view of communication, communicating information involves four basic components: the communication channel, the source (i.e. the person conveying the information), the audience (i.e. the person receiving the information) and the message (Carey, 2009). Accordingly, careful consideration of these components is pivotal for maximizing the impact of health campaigns (Rimal and Lapinski, 2009). Previous research on health campaigns highlighted variations in outcomes, possibly resulting from various characteristics of these components. For instance, in line with exemplification theory (Zillmann, 1999), research has shown that health campaigns involving exemplars (i.e. persons with lived experience of certain health conditions) as the communication source lead to stronger persuasion outcomes, compared to those without exemplars (Bigsby *et al*., 2019). This forms the foundation for inviting social media disclosers with lived experience of mental health problems as ‘health messengers’. Moreover, a substantial amount of literature has focused on the varying effects of audience characteristics on a range of health-related attitudinal and behavioral outcomes among the audience. For example, in the context of celebrity health disclosures and events (e.g. death by suicide, death by cancer), a meta-analysis found that audience members were more likely to modify their behavioral intentions toward the advocacy relevant to the health disclosures/events when they felt a sense of attachment or affinity with the celebrities (Kresovich and Noar, 2020). Likewise, survey studies (Lee *et al*., 2021; Myrick *et al*., 2022) and web-based data mining studies (Francis, 2021; Naik *et al*., 2021) indicated that similarity (both perceived and actual similarity, such as belonging to the same ethnic group) between audience members and celebrities plays a role in differentiating the effects of health-related disclosures/events across various groups of audiences.

However, in the specific context of public disclosures of mental health problems on *social media*, there is a lack of evidence underpinning how, when and which disclosures are more likely to be effective in stigma reduction (Gronholm and Thornicroft, 2022). Our recent systematic review synthesized research evidence for public disclosures of mental health problems on social media and their effects on stigma toward people with mental health problems (Zhang *et al*., 2024). The review described various disclosers, message features, audience characteristics, dynamics between disclosers and audiences, and the social media platforms on which the disclosures happened. More importantly, the review recommended that future research directly measure and monitor the anti-stigma effects of mental health-related disclosures on social media through collecting or linking ‘real’ data. Hence, the current study seeks to investigate the characteristics of disclosers, audience members and disclosure messages in public disclosures of mental health problems on social media, which occurred in real life rather than in an experimental setting.

The primary aim of this study was to explore whether there is any difference in audience perceptions of their own stigma reduction (referred to hereafter as audience self-reported stigma reduction) according to the various characteristics of these components. Particularly, this study focused on public stigma in relation to individuals with mental health problems. Utilizing a cross-sectional online survey design, this study addresses the following four research questions:

RQ1. Is there any difference in audience self-reported stigma reduction across different social media disclosers, according to (i) whether the discloser is a ‘traditional’ celebrity, ‘ordinary’ person or ‘emerging’ social media influencer? (ii) the disclosed mental health diagnoses?

RQ2. Is there an association between audience self-reported stigma reduction and the audience members’ (i.e. the participants’) perceived relationship with the disclosers?

RQ3. Is there any difference in audience self-reported stigma reduction according to the disclosure message content and format?

RQ4. Is there an association between audience self-reported stigma reduction and the characteristics of the online responses to the disclosure messages?

## METHODS

This study was preregistered with the Open Science Framework (Zhang, 2023) and received ethics approval from the University of Melbourne Human Research Ethics Committee (Ethics approval ID: 27844). Data were collected in December 2023.

### Participants, procedures and sample size estimation

Participants were recruited from Prolific (https://www.prolific.com/), an online crowdsourcing platform for researchers to launch studies to prescreened participants. Research has indicated that compared to other similar platforms, Prolific can provide consistently high-quality data in a cost-effective way (Peer *et al*., 2022). To obtain a custom sample of participants who encountered public disclosures of mental health problems on social media during the past year, we took a two-stage recruitment approach in accordance with the regulations of Prolific (2024). First, using the embedded prescreeners within Prolific, we posted a brief screening survey inviting potential participants who met the inclusion criteria: (i) aged 18 and above; (ii) proficient in English; (iii) had an approval rate of at least 90% and had completed at least 50 studies on Prolific. Participants answered ‘Yes’ or ‘No’ to four statements about their past-year social media activities. Participants who answered ‘Yes’ to the statement ‘Within the past year, I have seen people share their personal experiences of mental health problems publicly on social media’ entered the second stage of recruitment. We then sent these participants the invitation link to the main survey hosted on Qualtrics (https://www.qualtrics.com/au/) via their Prolific IDs. Participants were able to read through the plain language statement after clicking the link to the main survey, which they could access after they gave their informed consent to participation. Participants received a reimbursement payment via PayPal equivalent to GBP9/hour for their participation. Two attention-check questions were embedded into the main survey. Participants who failed both questions were rejected for a reimbursement payment and were excluded from further analyses. Relevant free public mental health helplines were offered at the end of the survey in case participants experienced any discomfort or distress from participation.

Previous literature indicated effect sizes ranging from Cohen’s *d* = 0.03 to 0.62 for anti-stigma effects of exposure to public disclosures of mental health problems (Miles, 2016; Taniguchi and Glowacki, 2021; Corrigan *et al*., 2022; Lotun *et al*., 2022). However, most of the effect sizes were around 0.20. According to GPower (Faul *et al*., 2007), a sample size of 828 for the main survey would give ~80% power to detect a small effect size of *d* = 0.20 with a two-sided 5% level of significance and would allow for 5% of responses to fail the two attention-check questions.

### Measures

The main survey included questions regarding participants’ demographic characteristics, social media usage, perceptions of public disclosures of mental health problems on social media, perceived relationship with social media disclosers, personal experiences in relation to mental health problems and self-reported stigma reduction attributable to seeing public disclosures of mental health problems on social media (see Supplementary Materials—online survey).

#### Demographic characteristics

Participants reported their age, gender, marital status, highest level of education and country of residence. As Prolific has a global pool of participants, we classified responses to country of residence according to country income level (The World Bank, 2024).

#### Social media usage

Participants reported their social media usage habits, including the platform(s) they regularly used and the approximate time duration they spent on social media daily.

#### Perceptions of public disclosures of mental health problems on social media

Participants were asked to reflect on their experiences of encountering public disclosures of mental health problems on social media within the last year. If there was more than one encounter, they were instructed to think about the one they remembered best. Participants categorized the social media discloser as a ‘traditional’ celebrity, an ‘emerging’ influencer or an ‘ordinary’ person. Participants then reported the mental health diagnosis (if any) the person disclosed. Further questions covered the format and content of the public disclosure message. These were adapted from previous research on key messages to use in population-level campaigns to alleviate mental health-related stigma (Clement *et al*., 2010). Participants were asked to identify the themes that the messages aligned with. There were 11 message themes to choose from, such as anti-dangerousness messages (i.e. ‘Countering stereotypes linking violence and mental health problems—for example, “I don’t think having mental health problems means not behaving well”’.) and social inclusion/human rights messages (i.e. ‘Advocating for the rights of people with mental health problems—for example, “I firmly believe that people with mental health problems have the right to the support they need to live their lives”’.). One more option was given to participants to allow them to report any themes apart from the 11 ones in a free-text format (i.e. ‘Other, please give details’). Participants also indicated whether there were any online comments/replies to the disclosure message and rated the overall valence of the comments on a scale from 0 (negative/stigmatizing) to 10 (positive/supportive). In addition, another open-ended question invited participants to provide more details on the content and context of the disclosures unless they selected ‘I can’t remember’ in the previous question regarding message themes (i.e. ‘Can you tell us any more information about the content of the disclosure message?’).

#### Perceived relationship with social media disclosers

To measure participants’ perceived relationship with social media disclosers, we specified three distinct constructs: empathy, perceived similarity and identification, based on the theory of identification with media characters (Cohen, 2001). Empathy was assessed by asking participants to rate their feelings toward the disclosers with reference to six adjectives (sympathetic, compassionate, soft-hearted, warm, tender, moved) (Batson, 2014) and also by asking participants to indicate the extent to which they empathized with the disclosers using one single item. Responses to these seven items were on a 7-point scale (1 = not at all, 4 = unsure, 7 = very much). An exploratory factor analysis utilizing the principal axis factoring extraction method demonstrated that the seven items could be aggregated into one single factor representing participants’ levels of empathy with the disclosers (internal consistency: McDonald’s omega = 0.93, 95% confidence interval [CI: 0.92, 0.94]) (Dunn *et al*., 2014). Therefore, the mean of the seven items was calculated, with higher scores representing higher levels of empathy with the disclosers. Perceived similarity and identification were gauged by asking participants to indicate the extent to which they perceived themselves as similar to or identified with the disclosers, each on a 7-point scale item (1 = not at all, 4 = unsure, 7 = very much). Higher scores indicate participants’ greater perceived similarity to or identification with the disclosers.

#### Personal experiences in relation to mental health problems

We modified the Level of Contact Report (Holmes *et al*., 1999) to capture participants’ own experiences with mental health problems. The Level of Contact Report has 12 items, which represent varying levels of contact with a person with a mental health problem. Items were ordered from the least intimate contact (i.e. ‘I have never observed a person that I was aware had a mental health problem’) to the most intimate contact (i.e. ‘I have a mental health problem’). Higher rank scores indicate closer contact. Interrater reliability has been shown to be high (0.83) (Holmes *et al*., 1999). The measure is scored by taking the highest rank score endorsed by expert consensus. We only used 10 of the 12 items in our study to avoid over-representations of social relationships indicated in the original report. Thus, participants’ scores ranged from 1 to 10. Previous research has shown that higher scores on the report correlate with less stigmatizing attitudes toward people with mental illness, demonstrating adequate validity (Corrigan *et al*., 2001).

### Audience self-reported stigma reduction

The Depression Stigma Scale (Griffiths *et al*., 2004) was adapted to measure audience/participant self-reported stigma reduction resulting from seeing public disclosures of mental health problems on social media. Prior literature has suggested that the Depression Stigma Scale has adequate psychometric properties (Yap *et al*., 2014). We only used the nine-item personal stigma subscale which assesses a participant’s own attitudes toward individuals with the same mental health problem as the discloser (e.g. ‘People with a problem like the person’s, i.e. the social media discloser, could snap out of it if they wanted’). The instructions and response scale were adapted for this study to measure audience self-reported stigma reduction out of exposure to public disclosures. Participants were presented with the lead-in before they proceeded to the nine statements ‘Compared to the time before you saw the disclosure, you are now more or less likely to agree that…’. Ratings were made on a five-point scale (1 = much less likely to agree, 2 = less likely to agree, 3 = no change, 4 = more likely to agree, 5 = much more likely to agree). Responses of scores 1 and 2 were then categorized into ‘stigma reduction’ (assigned a value of 1), while responses of scores 3, 4 and 5 were classified into ‘other’ (assigned a value of 0). An exploratory factor analysis based on principal axis factoring extraction and oblimin rotation method suggested a two-factor solution as found in previous research: ‘Weak-not-sick stigma’ and ‘Dangerous/unpredictable stigma’ (Yap *et al*., 2014). Scores for each factor were obtained by summing corresponding items in the factor. As the distributions were either highly skewed or bimodal, factor scores were further dichotomized to indicate any self-reported stigma reduction (scores of 1 or more) or no stigma reduction (scores of 0). Internal consistency scores in our sample were good for both factors (‘Weak-not-sick stigma’: McDonald’s omega = 0.80, 95% CI [0.78, 0.82]; ‘Dangerous/unpredictable stigma’: McDonald’s omega = 0.82, 95% CI [0.80, 0.83]).

Additionally, the five-item Social Distance Scale (Link *et al*., 1999) was modified to assess participants’ willingness to have contact with a person with a problem similar to the discloser in various social settings (e.g. ‘Move next door to people with a problem like the person’s, i.e. the social media discloser’). A lead-in was presented before the five items ‘Compared to the time before you saw the disclosure, you are now more or less willing to…’. Ratings were adjusted to a five-point scale, ranging from 1 = much less willing, to 3 = no change, to 5 = much more willing. Similarly, responses of scores 4 and 5 were then categorized into ‘stigma reduction’ (assigned a value of 1), while responses of scores 1, 2 and 3 were classified into ‘other’ (assigned a value of 0). Employing the principal axis factoring extraction method, an exploratory factor analysis indicated a unidimensional factor structure as advised in prior literature (McDonald’s omega = 0.86, 95% CI [0.84, 0.87]) (Yap *et al*., 2014). Scores were obtained through summing all five items. Likewise, scores were further dichotomized due to a highly skewed distribution, with scores of 0 allocated a value of 0 and scores higher than 0 allocated a value of 1 (indicating the presence of self-reported stigma reduction).

### Data analysis

All quantitative analyses were performed using R Statistical Software (version 4.3.2) (R Core Team, 2023). We explored the relationship between disclosure-related characteristics and audience self-reported stigma reduction. For this purpose, a series of logistic regression models were fitted for each stigma outcome. Key disclosure-related characteristics included the discloser’s identity and diagnosis, participants’ perceived relationship with the disclosers, the message’s format and content, and online responses to the messages. Demographic characteristics, including participants’ age, gender, marital status, level of education, country of residence, social media time use and personal experiences in relation to mental health problems were controlled for when modeling multivariate logistic regressions for audience self-reported stigma reduction based on each disclosure-related characteristic.

Free-text responses about any additional information regarding the content and context of the disclosures were exported to an Excel spreadsheet for content analysis (Hsieh and Shannon, 2005). Two authors (Z.Z. and N.R.) initially coded a random sample of 100 responses independently. A draft codebook was formulated during this process. The first author (Z.Z.) then coded all the remaining responses using the draft codebook and revised the codebook as necessary. This iterative process resulted in a final summary of the content analysis, where we reported the distinct themes, explanations and example quotes, and the frequency count of each theme. All authors reviewed and agreed on the summary.

## RESULTS

We received 812 responses to the main survey from Prolific. Nine failed the attention checks and were thus excluded from further analyses, resulting in a final sample of 803. The sample was relatively young with a mean age of 32.86 and approximately half (50.56%) were male. Participants were predominantly unmarried (75.59%), tertiary educated (65.01%) and from high-income countries (75.22%). The majority of participants (82.69%) spent over 1 hour on social media daily and participants had a mean contact score of 7.65, indicating an overall close contact with a person with a mental health problem (see Table 1). YouTube (82.44%), Instagram (72.48%) and Facebook (67.25%) were the most regularly used platforms among participants. Additionally, the majority of participants reported stigma reduction attributable to public disclosures of mental health problems on social media in weak-not-sick stigma (74.97%) and dangerous/unpredictable stigma (78.33%), while a relatively smaller proportion of participants reported stigma reduction in social distance stigma (57.29%).

**Table 1:** Participant characteristics and univariate logistic regressions for audience self-reported stigma reduction

Characteristic	Total (*n* = 803)	Univariate logistic regressions, odds ratios (95% CI)		
		Weak-not-sick stigma reduction	Dangerous/unpredictable stigma reduction	Social distance stigma reduction
Age, *M* (SD) range	32.86 (10.80) 18–76	0.99 (0.97, 1.00)	**0.98 (0.96, 0.99)**	**0.98 (0.97, 0.99)**
Gender, *n* (%)				
Male	406 (50.56)	(reference group)		
Female/other	397 (49.44)	0.75 (0.55, 1.04)	0.92 (0.65, 1.28)	0.81 (0.61, 1.07)
Marital status, *n* (%)				
Unmarried	607 (75.59)	(reference group)		
Married, de facto	196 (24.41)	1.00 (0.69, 1.45)	1.02 (0.69, 1.51)	0.75 (0.55, 1.04)
Education, *n* (%)				
Bachelor’s degree or above	522 (65.01)	(reference group)		
Below bachelor’s degree	281 (34.99)	**1.56 (1.13, 2.17)**	1.33 (0.94, 1.88)	1.09 (0.82, 1.46)
Country of residence, *n* (%)				
High-income countries	604 (75.22)	(reference group)		
Middle-income countries	199 (24.78)	**5.05 (2.94, 8.66)**	**4.76 (2.69, 8.44)**	**3.33 (2.31, 4.82)**
Contact score, *M* (SD) range	7.65 (2.49) 1–10^a^	**0.91 (0.85, 0.97)**	0.96 (0.90, 1.03)	1.01 (0.96, 1.07)
Social media time use daily, *n* (%)				
Less than 1 hour	139 (17.31)	(reference group)		
1–2 hours	209 (26.03)	1.52 (0.96, 2.43)	**1.76 (1.09, 2.84)**	**1.83 (1.18, 2.83)**
2–3 hours	200 (24.91)	**1.68 (1.04, 2.70)**	**2.04 (1.25, 3.35)**	**2.03 (1.31, 3.15)**
3–5 hours	142 (17.68)	**1.95 (1.15, 3.31)**	**2.51 (1.43, 4.41)**	**2.82 (1.73, 4.58)**
5 hours or more	113 (14.07)	**3.07 (1.65, 5.73)**	**3.10 (1.64, 5.84)**	**2.84 (1.69, 4.76)**

Bold values indicate *p* < 0.05.

^a^Higher scores indicate closer contact.

A series of univariate logistic regressions were conducted to explore whether there was any difference in audience self-reported stigma reduction across various demographic characteristics. The results indicated participants of older age were significantly less likely to report dangerous/unpredictable stigma reduction (odds ratio [OR] = 0.98, 95% CI [0.96, 0.99]) and social distance stigma reduction (OR = 0.98, 95% CI [0.97, 0.99]). Likewise, participants with closer contact with a person with a mental health problem were significantly less likely to report weak-not-sick stigma reduction (OR = 0.91, 95% CI [0.85, 0.97]). Conversely, participants having qualifications below a bachelor’s degree were significantly more likely to report weak-not-sick stigma reduction (OR = 1.56, 95% CI [1.13, 2.17]). Additionally, participants living in middle-income countries and those spending more time on social media daily were significantly more likely to report reduced stigma across all three mental health-related stigma measures (see Table 1).

### Social media disclosers

Over half of the participants (54.05%) reported seeing public disclosures of mental health problems from ‘ordinary’ people on social media (see Table 2). Depression (46.95%) and anxiety (20.05%) were the most frequently mentioned diagnoses by the nominated social media disclosers.

**Table 2:** Social media discloser characteristics and multivariate logistic regressions for audience self-reported stigma reduction

Characteristic	Total (*n* = 803)	Multivariate logistic regressions^a^, odds ratios (95% CI)		
		Weak-not-sick stigma reduction	Dangerous/unpredictable stigma reduction	Social distance stigma reduction
Social media discloser identity, *n* (%)				
An ‘ordinary’ person	434 (54.05)	(reference group)		
A ‘traditional’ celebrity	176 (21.92)	1.12 (0.73, 1.73)	1.02 (0.66, 1.61)	1.14 (0.79, 1.66)
An ‘emerging’ influencer	193 (24.03)	1.10 (0.72, 1.69)	1.00 (0.64, 1.58)	1.20 (0.83, 1.74)
Mental health diagnosis, *n* (%)				
I can’t remember	85 (10.59)	(reference group)		
No	91 (11.33)	1.42 (0.70, 2.91)	1.39 (0.70, 2.74)	1.34 (0.72, 2.50)
Yes^b^	627 (78.08)	1.08 (0.63, 1.81)	**1.85 (1.09, 3.08)**	**2.30 (1.42, 3.76)**
Mentions of anxiety diagnosis, *n* (%)	161 (20.05)	1.36 (0.87, 2.17)	1.30 (0.81, 2.15)	1.17 (0.80, 1.73)
Mentions of depression diagnosis, *n* (%)	377 (46.95)	1.11 (0.74, 1.64)	1.18 (0.77, 1.80)	1.17 (0.83, 1.65)
Mentions of eating disorders, *n* (%)	32 (3.99)	0.81 (0.36, 1.94)	0.72 (0.31, 1.82)	0.95 (0.45, 2.08)
Mentions of bipolar disorder diagnosis, *n* (%)	48 (5.98)	0.96 (0.48, 2.00)	0.83 (0.41, 1.82)	1.15 (0.61, 2.21)
Mentions of personality disorders, *n* (%)	23 (2.86)	2.64 (0.85, 11.67)	2.66 (0.74, 17.03)	0.99 (0.41, 2.49)
Mentions of neurodiverse conditions, *n* (%)	23 (2.86)	1.11 (0.43, 3.12)	1.59 (0.54, 5.83)	1.13 (0.46, 2.89)

Bold values indicate *p* < 0.05.

^a^Participant characteristics were controlled for, including age, gender, marital status, education, country of residence, contact score and social media time use.

^b^When directly compared to ‘No’, nonsignificant results for weak-not-sick stigma and dangerous/unpredictable stigma: 0.76 (0.43, 1.31), 1.33 (0.77, 2.27); significant results for social distance stigma: 1.72 (1.07, 2.77).

The identity of the social media disclosers (celebrity, influencer or ‘ordinary’ person) was not associated with any reduction of self-reported weak-not-sick stigma, dangerous/unpredictable stigma or social distance stigma. Nor were any of the specific mental health diagnoses the disclosers mentioned (anxiety, depression, eating disorders, bipolar disorder, personality disorders and neurodiverse conditions). Nearly 10% of disclosers mentioned some other diagnoses or conditions such as schizophrenia, post-traumatic stress disorder and suicide-related conditions. However, these were too mixed to warrant any regression analysis. Compared to participants who could not remember whether the disclosers explicitly mentioned any diagnosis, participants who were aware of disclosers’ diagnoses were significantly more likely to report reduced dangerous/unpredictable stigma (OR = 1.85, 95% CI [1.09, 3.08]) and social distance stigma (OR = 2.30, 95% CI [1.42, 3.76]), after controlling for demographic characteristics and social media time use. In addition, these participants were also significantly more likely to report reduced social distance stigma when directly compared to those who reported the disclosers did not mention any diagnosis (OR = 1.72, 95% CI [1.07, 2.77]).

### Relationship between disclosers and audiences/participants

As shown in Table 3, nearly 70% of the participants followed the social media accounts of the disclosers. Multivariate logistic regression models indicated that compared to participants who did not follow the disclosers’ accounts, those who followed were significantly more likely to report reduced dangerous/unpredictable stigma (OR = 1.72, 95% CI [1.15, 2.54]) and social distance stigma (OR = 1.48, 95%CI [1.05, 2.09]). Table 3 also presents the overall levels of participants’ empathy, perceived similarity and identification with the disclosers. After controlling for other participant characteristics, participants with higher levels of empathy toward, perceived similarity to, and identification with the disclosers were significantly more likely to report reduced mental health-related stigma (see Table 3). The exception was that perceived similarity was not significantly associated with any mitigation of weak-not-sick stigma.

**Table 3:** Relationship between disclosers and audience/participants and multivariate logistic regressions for audience self-reported stigma reduction

Characteristic	Total (*n* = 803)	Multivariate logistic regressions^a^, odds ratios (95% CI)		
		Weak-not-sick stigma reduction	Dangerous/unpredictable stigma reduction	Social distance stigma reduction
Have you (audience) ever followed the social media account of the discloser, *n* (%)				
No	203 (25.28)	(reference group)		
Yes	562 (69.99)	1.41 (0.96, 2.08)	**1.72 (1.15, 2.54)**	**1.48 (1.05, 2.09)**
I can’t remember	38 (4.73)	1.09 (0.50, 2.52)	0.99 (0.45, 2.26)	1.27 (0.62, 2.63)
Audience empathy towards discloser, *M* (SD) range	5.32 (1.18) 1–7^b^	**1.20 (1.04, 1.38)**	**1.42 (1.23, 1.65)**	**1.53 (1.33, 1.76)**
Audience perceived similarity with discloser, *M* (SD) range	3.67 (1.73) 1–7	1.09 (0.98, 1.20)	**1.12 (1.01, 1.25)**	**1.27 (1.16, 1.40)**
Audience identification with discloser, *M* (SD) range	3.90 (1.71) 1–7	**1.17 (1.05, 1.29)**	**1.21 (1.09, 1.35)**	**1.31 (1.20, 1.44)**

Bold values indicate *p* < 0.05.

^a^Participant characteristics were controlled for, including age, gender, marital status, education, country of residence, contact score and social media time use.

^b^Higher scores indicate higher levels of empathy, similarity, or identification.

We further examined whether empathy, similarity and identification varied according to the identity of the discloser (celebrity, influencer or ‘ordinary’ person). As indicated in Table S1, there were no significant differences in levels of empathy among participants exposed to these three different groups of disclosers. However, significantly higher levels of perceived similarity (*p* < 0.01) and identification (*p* < 0.01) were reported by participants who nominated ‘ordinary’ person disclosers, compared to those nominating celebrity disclosers. Therefore, we investigated whether the identity of the disclosers moderated the effects of empathy, perceived similarity and identification by adding interaction terms in logistic regression models for audience self-reported stigma reduction. We found the effects of empathy, perceived similarity and identification did not change according to the discloser’s identity.

### Social media disclosure messages

Instagram (32.50%), Facebook (25.16%), YouTube (19.55%) and X/Twitter (19.43%) were the social media platforms on which participants most commonly saw public disclosures of mental health problems. The most frequently used message formats were text (61.02%), video (43.84%) and picture/image (25.53%). The most commonly seen message contents involved the negative impact of mental illness (39.35%), encouraging help-seeking (32.25%) and psychosocial causes of mental ill health (31.76%) (referred to hereafter as psychosocial messages) (see Table 4). Results from the content analysis of 540 free-text responses largely echoed these findings regarding the content of the disclosure messages. Additional noteworthy themes that were not covered by the listed 11 ones from literature included disclosure of the time duration/trajectory of the mental health problem(s), disclosure of coping, disclosure of any stigma-related experience and provision of mental health-related knowledge. However, these extra themes were relatively infrequent (see Supplementary Materials—summaries of content analysis).

**Table 4:** Social media disclosure message characteristics and multivariate logistic regressions for audience self-reported stigma reduction

Characteristic	Total (*n* = 803)	Multivariate logistic regressions^a^, odds ratios (95% CI)		
		Weak-not-sick stigma reduction	Dangerous/unpredictable stigma reduction	Social distance stigma reduction
Social media disclosure message format^b^, *n* (%)				
Other	6 (0.75)			
I can’t remember	26 (3.24)			
Text	490 (61.02)	0.95 (0.66, 1.35)	1.29 (0.89, 1.86)	0.86 (0.63, 1.16)
Picture, image	205 (25.53)	1.23 (0.84, 1.82)	**1.53 (1.02, 2.34)**	**0.68 (0.48, 0.95)**
Video	352 (43.84)	1.24 (0.87, 1.77)	0.97 (0.67, 1.41)	**1.47 (1.08, 2.00)**
Reposting	56 (6.97)	1.40 (0.69, 3.08)	2.08 (0.92, 5.60)	1.04 (0.59, 1.90)
External links to relevant mental health services/education	45 (5.60)	1.11 (0.54, 2.40)	2.60 (1.08, 7.78)	1.69 (0.89, 3.36)
Social media disclosure message content, *n* (%)				
I can’t remember	85 (10.59)	(reference group)		
Any content topic	718 (89.41)	**1.88 (1.12, 3.13)**	**2.64 (1.58, 4.37)**	**2.11 (1.31, 3.44)**
Mentions of negative impact of mental illness messages, *n* (%)	316 (39.35)	1.37 (0.97, 1.94)	1.42 (0.99, 2.06)	1.16 (0.86, 1.57)
Mentions of encouraging help-seeking messages, *n* (%)	259 (32.25)	1.38 (0.96, 2.01)	**2.05 (1.37, 3.13)**	**1.48 (1.08, 2.04)**
Mentions of psychosocial messages, *n* (%)	255 (31.76)	**1.80 (1.23, 2.68)**	**1.76 (1.18, 2.67)**	**1.61 (1.17, 2.22)**
Mentions of social inclusion/human rights messages, *n* (%)	192 (23.91)	1.07 (0.72, 1.62)	**1.59 (1.02, 2.51)**	1.25 (0.88, 1.79)
Mentions of biomedical messages, *n* (%)	170 (21.17)	0.89 (0.59, 1.35)	0.95 (0.62, 1.49)	1.24 (0.86, 1.81)
Mentions of recovery-oriented messages, *n* (%)	145 (18.06)	1.33 (0.86, 2.11)	1.55 (0.97, 2.58)	1.46 (0.99, 2.18)
Mentions of see the person messages, *n* (%)	122 (15.19)	1.24 (0.77, 2.03)	1.67 (0.99, 2.94)	1.17 (0.78, 1.78)
Mentions of continuum messages, n (%)	85 (10.59)	1.27 (0.74, 2.29)	1.14 (0.65, 2.09)	1.32 (0.81, 2.16)
Mentions of valuing difference messages, *n* (%)	84 (10.46)	1.46 (0.83, 2.66)	1.61 (0.89, 3.13)	1.30 (0.80, 2.13)
Mentions of anti-dangerousness messages, *n* (%)	73 (9.09)	1.74 (0.94, 3.45)	**2.17 (1.09, 4.84)**	1.12 (0.67, 1.89)
Mentions of high prevalence of mental disorders messages, *n* (%)	57 (7.10)	1.50 (0.75, 3.21)	1.77 (0.84, 4.22)	1.33 (0.74, 2.45)

Bold values indicate *p* < 0.05.

^a^Participant characteristics were controlled for, including age, gender, marital status, education, country of residence, contact score and social media time use.

^b^Numbers do not add to 100% as multiple responses allowed.

Messages containing pictures/images were significantly less likely to be associated with reduced social distance stigma when controlling for other participant characteristics (OR = 0.68, 95% CI [0.48, 0.95]). However, these messages were significantly more likely to be associated with reduced dangerous/unpredictable stigma when controlling for other participant characteristics (OR = 1.53, 95% CI [1.02, 2.34]). Similarly, video messages were significantly more likely to be associated with reduced social distance stigma in the multivariate model (OR = 1.47, 95%CI [1.08, 2.00]).

Compared to participants who did not remember the content of the messages, participants who recalled any content of the messages were more likely to report reduced mental health-related stigma across all three measures, after controlling for other participant characteristics. Among the specific topics of the messages, mentions of psychosocial messages were significantly more likely to be associated with participants’ self-reported stigma reduction across all three measures in the multivariate models. Similarly, messages encouraging help-seeking were significantly more likely to be associated with reduced dangerous/unpredictable stigma and social distance stigma. In addition, mentions of anti-dangerousness messages and social inclusion/human rights messages were significantly more likely to be associated with reduced dangerous/unpredictable stigma when controlling for other participant characteristics.

### Online comments to disclosure messages

As shown in Table 5, approximately half of the participants (51.06%) observed comments/replies from other social media users to the disclosure messages. These 410 participants reported an overall positive valence of the observed comments (Mean valence = 8.19, standard deviation [SD] = 1.87). Specifically, 285/410 (69.51%) participants reported seeing similar public disclosures of mental health problems in other social media users’ comments to the disclosure messages. Logistic regression models indicated that the presence/absence of online comments to disclosure messages was not associated with any reduction in mental health-related stigma. However, when comments were present, those with a more positive valence and those containing (many) similar disclosures were more likely to be associated with reduced social distance stigma when controlling for other participant characteristics (see Table 5).

**Table 5:** Social media comments to disclosure messages and multivariate logistic regressions for audience self-reported stigma reduction

Characteristic	Total (*n* = 803)	Multivariate logistic regressions^a^, odds ratios (95% CI)		
		Weak-not-sick stigma reduction	Dangerous/unpredictable stigma reduction	Social distance stigma reduction
Comments/replies to social media disclosure message, *n* (%)				
I can’t remember	204 (25.40)	(reference group)		
No	189 (23.54)	1.09 (0.68, 1.74)	0.95 (0.59, 1.54)	1.02 (0.67, 1.54)
Yes	410 (51.06)	1.32 (0.88, 1.97)	1.33 (0.87, 2.03)	1.20 (0.84, 1.71)
Subtotal (*n* = 410)				
Comments positivity/negativity valence, *M* (SD) range	8.19 (1.87) 0–10^b^	1.00 (0.87, 1.14)	1.04 (0.90, 1.19)	**1.15 (1.03, 1.30)**
Disclosures in comments from other social media users, *n* (%)				
I can’t remember	86 (20.98)	(reference group)		
None	39 (9.51)	1.49 (0.57, 4.27)	1.34 (0.51, 3.87)	1.08 (0.49, 2.38)
Some	215 (52.44)	1.09 (0.57, 2.02)	1.01 (0.52, 1.92)	1.49 (0.88, 2.56)
Many	70 (17.07)	0.96 (0.43, 2.14)	1.81 (0.74, 4.68)	**2.92 (1.45, 6.06)**

Bold values indicate *p* < 0.05.

^a^Participant characteristics were controlled for, including age, gender, marital status, education, country of residence, contact score and social media time use.

^b^Higher scores indicate higher levels of positivity valence.

## DISCUSSION

This online survey study explored variations in audience perceptions of their own stigma reduction attributable to exposure to public disclosures of mental health problems on social media. The study considered three components in such disclosures: the social media disclosers, the audiences/participants who saw the disclosures and the disclosure messages. Findings suggest that explicit diagnoses from social media disclosers, mentions of particular message themes such as psychosocial causes, and the presence of positive and echoing comments from other users are most likely to be associated with audience self-reported stigma reduction. In addition, when audience members report greater levels of empathy toward, perceived similarity to and identification with the disclosers, they tend to report reduced stigma toward people with mental health problems.

### Audience segmentation

Health-related disclosures can affect segments of the population differently, according to their demographic characteristics and personal connections with the disclosers (Myrick *et al*., 2024). The current study found that audience members from middle-income countries and those with lower levels of educational attainment were more likely to report reduced mental health-related stigma, compared to their counterparts. These findings appear plausible, given that surveys of mental illness stigma suggest relatively lower levels of stigma among individuals from high-income countries (Pescosolido *et al*., 2015; Seeman *et al*., 2016) and those who are more educated (Corrigan and Watson, 2007). Therefore, there may be ceiling effects that make it more challenging for these particular audiences to report further stigma reduction from such brief exposures. Previous research has reported on the relatively larger proportions of less educated populations living in under-resourced areas (Barro and Lee, 2013), and the relative paucity of mainstream media reports of lived experience stories related to mental ill health (Armstrong *et al*., 2023). Our findings lend evidence to addressing mental health stigma in under-resourced settings through digital mental health interventions on social media (Naslund and Deng, 2021).

Furthermore, the results revealed that younger audience members, individuals with less prior contact with people with mental health problems and those who spent more time on social media were more likely to report reduced stigma. These findings highlight that although social media use has been found to be associated with mental health vulnerabilities, especially among young people (Orben *et al*., 2024), it can encourage positive health-related attitudinal and behavioral changes by creating opportunities for contact with minority groups (a process grounded in intergroup contact theory (Allport, 1954; Pettigrew and Tropp, 2006)). Nevertheless, it should be noted that this study did not involve participants below the age of 18. These younger people make up a substantial portion of users on more recent social media platforms such as TikTok, which more frequently feature ‘emerging’ influencers and catalyze online discourses (DataReportal, 2023). This warrants future research to specifically investigate the effects of online public disclosures of mental health problems on these platforms on younger people.

### Protagonist in health messaging

As protagonists, social media disclosers exhibit various characteristics, which potentially contribute to variations in health messaging outcomes. Our survey found that disclosers’ mentioning any specific diagnosis in the message was significantly associated with audience self-reported stigma reduction, especially with social distance stigma reduction. Previous research has suggested that labeling has both positive and negative effects on addressing mental health stigma (Link and Phelan, 2009; Wright *et al*., 2011). In our study, exposure to disclosure messages with specific diagnoses created opportunities for contact with individuals with those mental health problems among the audience members and thus may have had a positive effect on stigma reduction.

In addition, the present study revealed the identity of the discloser (celebrity, influencer or ‘ordinary’ person) was not associated with any mitigation of mental health stigma among audience members. Instead, consistent with previous research (Gronholm and Thornicroft, 2022; Myrick *et al*., 2024), our findings highlighted the importance of the audience’s perceived relationship with disclosers in achieving expected health promotion effects. Moreover, we found that the effects of the audience’s perceived relationship with disclosers did not vary across different identity groups of disclosers. These findings extend the current evidence base regarding the role of celebrity/fame in health messaging, which has had mixed results. For instance, one experimental study by Corrigan *et al*. (2022) found that mental health-related public disclosures from non-celebrities led to greater reductions in stigma than those from celebrities, possibly as non-celebrities were perceived as more similar to audiences and more admirable. Conversely, though not related to mental health disclosures, another experimental study by E. Cohen (2020) found Tom Hanks’ disclosure of his coronavirus disease 2019 (COVID-19) infection significantly increased audience COVID-19-related risk perceptions, anxiety and intentions to engage in health-preventative behaviors, compared to disclosure from a non-celebrity. These effects remained significant even when audience personal involvement with Hanks (i.e. parasocial relationship and wishful identification) was controlled for. Consequently, E. Cohen (2020) argued that although both celebrity and non-celebrity disclosures may work as exemplars in health campaigns according to exemplification theory (Zillmann, 1999), the mere presence of Hanks’ celebrity, as a socialized symbol, might independently function as a cognitive and affective shortcut on which the audience can base their risk assessments and health behavior decision making. Nevertheless, our study indicates that the audience’s perceived relationship with disclosers, rather than disclosers’ identities, might be a more important contributor to variations in messaging outcomes.

Future research might enhance the current evidence in this regard. Specifically, it remains a challenge to clarify the conceptualization and operationalization of the audience’s perceived relationship with disclosers. In our study, we measured audience empathy toward, perceived similarity to and identification with disclosers in line with extant literature regarding identification with media figures (Cohen, 2001). However, prior studies have also used other proxies to evaluate the relationship between audiences and disclosers, such as parasocial relationship (Hoffner and Cohen, 2018; Lee *et al*., 2021), sense of attachment or affinity (Kresovich and Noar, 2020), and relational bond (Leung, 2019). Future research might benefit from theoretically distinguishing these various aspects in the dynamics between audiences and disclosers. More importantly, researchers may empirically examine which aspect matters most in the context of brief exposure to online health campaigns. Additionally, it is worth noting that among celebrities, the type of fame varies significantly (e.g. music, sports, politics or lifestyle) (Corrigan *et al*., 2022). This highlights the interplay of various protagonists’ attributes in health messaging. Future research may also consider whether there exist any pathways (e.g. any aspect in relation to the perceived relationship mentioned above) through which the identity of a celebrity or non-celebrity exerts impact on health-related campaign outcomes.

### Message in health campaigns

Previous research has found comparable anti-stigma effects across different formats used in mental health campaigns, including texts, videos, live performances and combinations of various materials (Maunder and White, 2019; Naslund and Deng, 2021; Makhmud *et al*., 2022). Nevertheless, our survey found that messages incorporating visual elements (e.g. pictures and videos) were more likely to be associated with audience self-reported stigma reduction. This speaks to the advantages visual information has in communication, such as enabling greater engagement with audiences (Li and Xie, 2020). Interestingly, there were some contradictory effects regarding the associations between visual elements and social distance reduction. Pictures were less likely to be associated with social distance stigma reduction, whereas videos were more likely to be associated with social distance stigma reduction. Therefore, future practices may consider utilizing more video-based messages for public disclosures of mental health problems on social media (e.g. vlogs). However, the specific content conveyed in those pictures or videos might be the key contributor to the contradictory findings.

Our findings suggested the specific content conveyed in the disclosure message was a factor in the audience self-reported stigma reduction. Based on the types of key messages used in population-level mental health stigma alleviation campaigns (Clement *et al*., 2010), our study showed that psychosocial messages were more likely to be associated with audience self-reported stigma reduction across three stigma measures. This is slightly different from previous research into ‘causal beliefs’ of mental health problems and their effects on stigma. For instance, Reavley and Jorm (2014) found inconsistent patterns of associations between belief in psychosocial causes and stigmatizing attitudes in a national survey of the Australian public. They further proposed that psychosocial messages may be not suitable for stigma mitigation campaigns. However, a recent scoping review synthesized existing research on causal beliefs about mental illness. The review recommended that research should contextualize their findings and should not assume that certain beliefs are universally helpful or harmful (Ahuvia *et al*., 2024).

In addition, our study found significant associations between mentions of encouraging help-seeking messages and audience self-reported stigma reduction. This provides evidence for current mental health promotion practices that usually involve elements of encouraging help-seeking. More importantly, we observed that anti-dangerousness messages were more likely to be associated with dangerous/unpredictable stigma reduction. This underscores the significance of understanding and addressing mental health-related stigma as a multifaceted construct (Yap *et al*., 2014). It also lends evidence to the usefulness of mitigating certain components of stigma through designing component-specific elements in anti-stigma campaign messages or balanced news reporting of mental illness in the context of violent crime (Morgan *et al*., 2022; Zhang *et al*., 2024).

Moreover, our findings suggested that when online comments to public disclosures of mental health problems were present, disclosure messages followed by comments with higher levels of positivity and more ‘copycat’ disclosures from other social media users were more likely to be associated with audience self-reported social distance stigma reduction. While moderating audience comments may pose certain ethical and practical concerns, these findings have critical implications. For example, they emphasize the role of supportive peer responses to disclosure messages on social media in reducing stigma among audiences. This is consistent with previous research (Taniguchi and Glowacki, 2021). Furthermore, similar disclosures in comments from other users allude to some reductions in these users’ stigmatizing perceptions of themselves (i.e. self-stigma). Future research might benefit from examining the effects of self-stigma of exposure to public disclosures on social media among audiences with mental health problems. These echoing disclosures, alongside the primary disclosure message, also highlight the importance of multiple exposures in health messaging (Hornik, 2016). However, there might be an ‘echo chamber’ around the disclosures on social media, where opinions or attitudes of users about a certain topic (i.e. mental health in this study) are confirmed and reinforced because of repeated interactions with peers having similar tendencies (i.e. pro-mental health in this study) (Cinelli *et al*., 2021). This could potentially limit the impact of the messages across populations. Future research should be conducted to investigate this phenomenon and explore ways to extend the message impact.

### Practical implications

The study has several practical implications for health promotion practices. First, based on the findings regrading audience segmentation, future health campaign efforts, particularly those in under-resourced areas, may consider incorporating public disclosures on social media to achieve greater reach and impact at a population level. Second, in order to maximize the positive effects of social media while minimizing any risks, health promotion practitioners may proactively work with social media users (especially young people), technology companies and policy-makers to create a safe and meaningful online ecosystem that enhances health promotion outcomes (Robinson *et al.*, 2023). Health promotion practitioners may also engage with social media users (especially those with larger numbers of followers such as celebrities and influencers) with mental health problems who are willing to publicly disclose their personal experiences online. Together, evidence-based guidelines could be developed to create effective online anti-stigma messaging. Similarly, guidelines around how to appropriately respond to online public disclosures could be developed. This proactive working approach is particularly to minimize the risks of misinformation being disseminated through disclosure messages and subsequent responses. For instance, Kumble *et al*. (2022) detected the existence of social media bots that deliberately disseminated stigmatizing sentiments in response to disclosures. Third, considering the significance of the dynamics between audiences and disclosers, future practices may benefit from carefully considering the match between the various attributes of social media disclosers and the intended audiences (Gronholm and Thornicroft, 2022). Last, instead of identifying a universally applicable suite of best practices, health promotion practitioners may situate health campaigns within specific cultural and contextual settings.

### Strengths and limitations

The study’s strengths included its focus on real-life disclosures of mental health problems on social media, rather than experimentally manipulated hypothetical vignette, and its utilization of the theoretical traditions in communication studies to design the study and interpret the results.

However, the study has some limitations. First, the cross-sectional design precludes any inferences of causality such as temporal connections between greater levels of empathy and reduced stigma. Probing participants’ attitudinal changes at one single time point with a survey lead-in ‘Compared to the time before you saw the disclosure…’ is less rigorous than doing pre–post assessments across different time points. Second, all data were self-reported by participants and thus social desirability and recall bias (especially the year-long recollection period) represent additional limitations. Future research may harness big digital behavioral data and possibly link them to other data sources to monitor and assess real-time audience responses in relation to stigma. Third, the survey questionnaire only collected a limited range of demographic variables. This restricts more granular analyses into health disparities across populations (e.g. disparities across various populations of different sexual orientations). In addition, the final sample does not include participants from low-income countries, limiting the ability to generalize to these settings. Last, the questionnaire in our survey limits robust explorations of whether the effects extend beyond the mental health condition(s) involved in the disclosure (Maunder and White, 2019).

## CONCLUSION

The present study suggests that certain aspects of the messaging process in public disclosures of mental health problems on social media appear to be associated with audience self-reported stigma reduction. These include explicit diagnoses from social media disclosers, the inclusion of certain message themes such as psychosocial causes of mental ill health, as well as positive and echoing comments from other users. These findings can help inform message designs in anti-stigma campaigns that incorporate public disclosures of mental health problems on social media. Future research is warranted to investigate the dynamics between disclosers and audience members and whether the transfer effects of diagnoses still apply in disclosure messages.

## Supplementary Material

daae204_suppl_Supplementary_File1

daae204_suppl_Supplementary_Tables_S1

daae204_suppl_Supplementary_File2

## Data Availability

The data that support the findings of this study are available from the corresponding author, Z.Z., upon reasonable request.
