# Supplementary material for: Public disclosures of mental health problems on social media and audiences’ self-reported anti-stigma effects
Source: Health Promot Int. 2025 Jan 21;40(1):daae204. doi: 10.1093/heapro/daae204 (PMC11747871; doi:10.1093/heapro/daae204)
Supplement: daae204_suppl_Supplementary_File1 [file daae204_suppl_supplementary_file1.docx]

Social media survey – screening survey

**Please answer “yes/no” to the following statements**

1. Within the past year, I have followed the TikTok accounts of at least two entertainment celebrities.
2. Within the past year, I have spent most of my spare time on YouTube to view videos about world geography.
3. Within the past year, I have seen people share their personal experiences of mental health problems publicly on social media.
4. Within the past year, I have seen lots of online advertisements on social media, which offered direct links to online shopping websites.

Social media survey – main survey

Start of Block: Demographics

**First, we want to know something about you 👫**

| 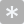 |
| --- |

What is your age?

________________________________________________________________

What is your gender?

- Male
- Female
- Other, please give details __________________________________________________

What is your current marital status?

- Never married
- Married, de facto
- Separated, divorced or widowed

What is the highest level of education you have completed?

- Primary/elementary school
- Secondary/high school
- Certificate, Trade or Apprenticeship
- Bachelor degree
- Postgraduate degree

What country do you live in?

- USA
- UK
- Australia
- Other, please give details __________________________________________________

End of Block: Demographics

Start of Block: Social media usage

**🧐 You might have at least 1️⃣ personal social media account. We now would like you to please tell us your social media usage habits.**

What social media platforms do you use regularly? - **multiple choices allowed**

- Facebook
- Twitter/X
- Instagram
- YouTube
- TikTok
- Other, please give details __________________________________________________

How much time do you typically spend on social media per day?

- None
- Less than half an hour
- 0.5 hour – 1 hour
- 1-2 hours
- 2-3 hours
- 3-5 hours
- 5-7 hours
- 7 hours or more

End of Block: Social media usage

Start of Block: Respondent’s own experiences in relation to mental health problems

**✍️ For the purpose of this project, by a ‘mental health problem’ we mean a period of weeks or more when a person is feeling depressed, anxious, or emotionally stressed, and these problems are interfering with their life. Mental health problems could include, for example, depression, anxiety disorders, eating disorders, schizophrenia, bipolar disorder, or personality disorders.**

**Next, please read each of the following statements carefully and select the statements that align with your own experiences - multiple choices allowed**

- I have watched a movie or television show in which a character depicted a person with a mental health problem.
- My job involves providing services/treatment for people with mental health problems.
- I have observed, in passing, a person I believe may have had a mental health problem.
- I have observed people with mental health problems on a frequent basis.
- I have a mental health problem.
- I have worked with a person who had a mental health problem at my place of employment.
- I have never observed a person that I was aware had a mental health problem.
- A friend of the family has a mental health problem.
- I have a relative who has a mental health problem.
- I live with a person who has a mental health problem.

End of Block: Respondent’s own experiences in relation to mental health problems

Start of Block: Perceptions of public disclosures of mental health problems on social media

**In our previous short survey, we noticed that you have seen someone publicly disclose their mental health problems on social media during the past year.

 👉Now please keep that person in mind 👀
 We'd like you to tell us more about this particular experience. 
 *Note if there is more than one person, please think about the one you remember best.***

Who is the person who publicly shared their experiences of mental health problems on social media?

- A “traditional” celebrity (such as a widely-recognised movie star, singer or athlete)
- An “emerging” influencer (such as a grassroots YouTuber, TikToker with a lot of followers)
- An “ordinary” person (such as your peer in real life, or another social media user who you do not know offline)

Did they disclose a specific mental health problem diagnosis? For example, depression, panic attack or eating disorders.

- Yes
- No
- I can't remember

Display This Question:

If Did they disclose a specific mental health problem diagnosis? For example, depression, panic atta... = Yes

| 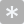 |
| --- |

What was this diagnosis?

________________________________________________________________

Display This Question:

If Who is the person who publicly shared their experiences of mental health problems on social media? = A “traditional” celebrity (such as a widely-recognised movie star, singer or athlete)

Who is the celebrity?

________________________________________________________________

Display This Question:

If Who is the person who publicly shared their experiences of mental health problems on social media? = A “traditional” celebrity (such as a widely-recognised movie star, singer or athlete)

Have you ever followed the social media account of the celebrity?

- Yes
- No
- I can't remember

Display This Question:

If Who is the person who publicly shared their experiences of mental health problems on social media? = A “traditional” celebrity (such as a widely-recognised movie star, singer or athlete)

On a scale of 1 to 7, please rate your feelings towards the celebrity in terms of the following six adjectives

|  | **1=Not at all** | **4=Unsure** | **7=Very much** |
| --- | --- | --- | --- |

|  | 1 | 2 | 3 | 4 | 5 | 6 | 7 |
| --- | --- | --- | --- | --- | --- | --- | --- |

| Sympathetic | 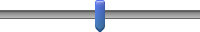 |
| --- | --- |
| Compassionate | 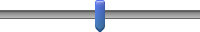 |
| Soft-hearted | 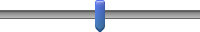 |
| Warm | 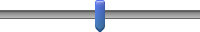 |
| Tender | 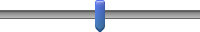 |
| Moved | 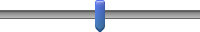 |

Display This Question:

If Who is the person who publicly shared their experiences of mental health problems on social media? = A “traditional” celebrity (such as a widely-recognised movie star, singer or athlete)

On a scale of 1 to 7, please indicate the extent to which you empathise with the celebrity

|  | **1=Not at all** | **4=Unsure** | **7=Very much** |
| --- | --- | --- | --- |

|  | 1 | 2 | 3 | 4 | 5 | 6 | 7 |
| --- | --- | --- | --- | --- | --- | --- | --- |

| Empathy | 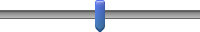 |
| --- | --- |

Display This Question:

If Who is the person who publicly shared their experiences of mental health problems on social media? = A “traditional” celebrity (such as a widely-recognised movie star, singer or athlete)

On a scale of 1 to 7, please indicate the extent to which you perceive yourself as similar to the celebrity

|  | **1=Not at all** | **4=Unsure** | **7=Very much** |
| --- | --- | --- | --- |

|  | 1 | 2 | 3 | 4 | 5 | 6 | 7 |
| --- | --- | --- | --- | --- | --- | --- | --- |

| Similarity | 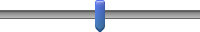 |
| --- | --- |

Display This Question:

If Who is the person who publicly shared their experiences of mental health problems on social media? = A “traditional” celebrity (such as a widely-recognised movie star, singer or athlete)

On a scale of 1 to 7, please indicate the extent to which you identify with the celebrity

|  | **1=Not at all** | **4=Unsure** | **7=Very much** |
| --- | --- | --- | --- |

|  | 1 | 2 | 3 | 4 | 5 | 6 | 7 |
| --- | --- | --- | --- | --- | --- | --- | --- |

| Identification | 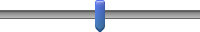 |
| --- | --- |

Display This Question:

If Who is the person who publicly shared their experiences of mental health problems on social media? = An “emerging” influencer (such as a grassroots YouTuber, TikToker with a lot of followers)

Who is the influencer?

________________________________________________________________

Display This Question:

If Who is the person who publicly shared their experiences of mental health problems on social media? = An “emerging” influencer (such as a grassroots YouTuber, TikToker with a lot of followers)

Have you ever followed the social media account of the influencer?

- Yes
- No
- I can't remember

Display This Question:

If Who is the person who publicly shared their experiences of mental health problems on social media? = An “emerging” influencer (such as a grassroots YouTuber, TikToker with a lot of followers)

On a scale of 1 to 7, please rate your feelings towards the influencer in terms of the following six adjectives

|  | **1=Not at all** | **4=Unsure** | **7=Very much** |
| --- | --- | --- | --- |

|  | 1 | 2 | 3 | 4 | 5 | 6 | 7 |
| --- | --- | --- | --- | --- | --- | --- | --- |

| Sympathetic | 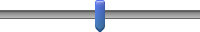 |
| --- | --- |
| Compassionate | 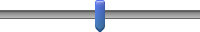 |
| Soft-hearted | 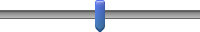 |
| Warm | 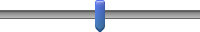 |
| Tender | 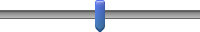 |
| Moved | 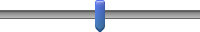 |

Display This Question:

If Who is the person who publicly shared their experiences of mental health problems on social media? = An “emerging” influencer (such as a grassroots YouTuber, TikToker with a lot of followers)

On a scale of 1 to 7, please indicate the extent to which you empathise with the influencer

|  | **1=Not at all** | **4=Unsure** | **7=Very much** |
| --- | --- | --- | --- |

|  | 1 | 2 | 3 | 4 | 5 | 6 | 7 |
| --- | --- | --- | --- | --- | --- | --- | --- |

| Empathy | 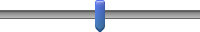 |
| --- | --- |

Display This Question:

If Who is the person who publicly shared their experiences of mental health problems on social media? = An “emerging” influencer (such as a grassroots YouTuber, TikToker with a lot of followers)

On a scale of 1 to 7, please indicate the extent to which you perceive yourself as similar to the influencer

|  | **1=Not at all** | **4=Unsure** | **7=Very much** |
| --- | --- | --- | --- |

|  | 1 | 2 | 3 | 4 | 5 | 6 | 7 |
| --- | --- | --- | --- | --- | --- | --- | --- |

| Similarity | 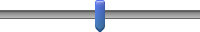 |
| --- | --- |

Display This Question:

If Who is the person who publicly shared their experiences of mental health problems on social media? = An “emerging” influencer (such as a grassroots YouTuber, TikToker with a lot of followers)

On a scale of 1 to 7, please indicate the extent to which you identify with the influencer

|  | **1=Not at all** | **4=Unsure** | **7=Very much** |
| --- | --- | --- | --- |

|  | 1 | 2 | 3 | 4 | 5 | 6 | 7 |
| --- | --- | --- | --- | --- | --- | --- | --- |

| Identification | 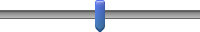 |
| --- | --- |

Display This Question:

If Who is the person who publicly shared their experiences of mental health problems on social media? = An “ordinary” person (such as your peer in real life, or another social media user who you do not know offline)

Do you know the person offline in real life?

- Yes
- No

Display This Question:

If Who is the person who publicly shared their experiences of mental health problems on social media? = An “ordinary” person (such as your peer in real life, or another social media user who you do not know offline)

Have you ever followed the social media account of the person?

- Yes
- No
- I can't remember

Display This Question:

If Who is the person who publicly shared their experiences of mental health problems on social media? = An “ordinary” person (such as your peer in real life, or another social media user who you do not know offline)

And Do you know the person offline in real life? = Yes

How would you describe your relationship with the person?

- Family member
- Intimate partner
- Friend
- Work colleague
- Other, please give details __________________________________________________

Display This Question:

If Who is the person who publicly shared their experiences of mental health problems on social media? = An “ordinary” person (such as your peer in real life, or another social media user who you do not know offline)

On a scale of 1 to 7, please rate your feelings towards the person in terms of the following six adjectives

|  | **1=Not at all** | **4=Unsure** | **7=Very much** |
| --- | --- | --- | --- |

|  | 1 | 2 | 3 | 4 | 5 | 6 | 7 |
| --- | --- | --- | --- | --- | --- | --- | --- |

| Sympathetic | 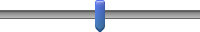 |
| --- | --- |
| Compassionate | 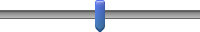 |
| Soft-hearted | 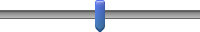 |
| Warm | 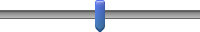 |
| Tender | 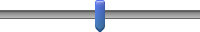 |
| Moved | 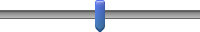 |

Display This Question:

If Who is the person who publicly shared their experiences of mental health problems on social media? = An “ordinary” person (such as your peer in real life, or another social media user who you do not know offline)

On a scale of 1 to 7, please indicate the extent to which you empathise with the person

|  | **1=Not at all** | **4=Unsure** | **7=Very much** |
| --- | --- | --- | --- |

|  | 1 | 2 | 3 | 4 | 5 | 6 | 7 |
| --- | --- | --- | --- | --- | --- | --- | --- |

| Empathy | 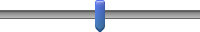 |
| --- | --- |

Display This Question:

If Who is the person who publicly shared their experiences of mental health problems on social media? = An “ordinary” person (such as your peer in real life, or another social media user who you do not know offline)

On a scale of 1 to 7, please indicate the extent to which you perceive yourself as similar to the person

|  | **1=Not at all** | **4=Unsure** | **7=Very much** |
| --- | --- | --- | --- |

|  | 1 | 2 | 3 | 4 | 5 | 6 | 7 |
| --- | --- | --- | --- | --- | --- | --- | --- |

| Similarity | 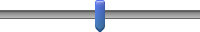 |
| --- | --- |

Display This Question:

If Who is the person who publicly shared their experiences of mental health problems on social media? = An “ordinary” person (such as your peer in real life, or another social media user who you do not know offline)

On a scale of 1 to 7, please indicate the extent to which you identify with the person

|  | **1=Not at all** | **4=Unsure** | **7=Very much** |
| --- | --- | --- | --- |

|  | 1 | 2 | 3 | 4 | 5 | 6 | 7 |
| --- | --- | --- | --- | --- | --- | --- | --- |

| Identification | 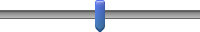 |
| --- | --- |

Which social media platform(s) did the person use to make their disclosure? – **multiple choices allowed**

- YouTube
- Twitter/X
- ⊗I can't remember
- Facebook
- Instagram
- TikTok
- Other, please give details __________________________________________________

What content did the disclosure message cover? - **multiple choices allowed**

- Biological causes of mental health problems (genetics, chemical imbalance), treatable by medication - For example: "I have been on medication for some time to manage my mental health symptoms"
- Many people experience mental health problems - For example: "In my daily life, it is common to hear about people experiencing some form of mental health problems "
- Countering stereotypes linking violence and mental health problems - For example: "I don’t think having mental health problems means not behaving well"
- ⊗I can't remember
- Difference can be positive, value of diversity, pride in experience, questioning “normality” - For example: "I started wondering what a normal person without abnormal mental health status would look like?"
- Advocating for the rights of people with mental health problems - For example: "I firmly believe that people with mental health problems have the right to the support they need to live their lives"
- Social/psychological causes of mental health problems - For example: “My mental health problem came after a significant life event – the loss of a loved one”
- Emphasising the person over the problems, seeing the whole person - For example: "It is true that I have schizophrenia, but it is also true that I am a person with both strengths and weaknesses"
- The boundaries between mental health and mental health problems are not clear-cut - For example: "Sometimes I am not sure whether those obsessive impulses are the symptoms which have troubled me a lot, or the personal traits that have benefited me in life and work"
- Living satisfying, hopeful and contributing lives despite problems - For example: "I am still living the life I have always dreamed of despite my mental health problem"
- Serious consequences of mental health problems - For example: "I have had a very difficult time coping with my symptoms"
- Providing advice on seeking relevant help and encouraging help-seeking – For example: “I strongly suggest that if you are feeling the way I was before, please do talk to a mental health professional”
- Other, please give details __________________________________________________

Display This Question:

If What content did the disclosure message cover? - multiple choices allowed != I can't remember

Can you tell us any more information about the content of the disclosure message?

________________________________________________________________

What format(s) did the disclosure message employ? – **multiple choices allowed**

- Text
- Picture, image
- Video
- ⊗I can't remember
- Quotes from other sources
- External links to relevant mental health services/education
- Other, please give details __________________________________________________

Did you notice any online comments/replies to the disclosure message?

- No
- Yes
- I can't remember

Display This Question:

If Did you notice any online comments/replies to the disclosure message? = Yes

Were the comments/replies mostly negative or positive? Please rate on a scale from 0 to 10.
 **0 = negative/stigmatising  5 = neutral  10 = positive/supportive**

- 0
- 1
- 2
- 3
- 4
- 5
- 6
- 7
- 8
- 9
- 10

Display This Question:

If Did you notice any online comments/replies to the disclosure message? = Yes

Were there any social media users who commented/replied to the disclosure message and publicly disclosed their own mental health problems in their comments/replies?

- ⊗I can't remember
- ⊗None
- Some
- Many

End of Block: Perceptions of public disclosures of mental health problems on social media

Start of Block: Anti-stigma impact perceptions

We are interested in **whether there was a significant change** in the way you perceived people with mental health problems **before and after** seeing the disclosure messages.

**Compared to the time before you saw the disclosure,** **[you are now more or less likely to agree that]**

|  | much less likely | less likely | no change | more likely | much more likely |
| --- | --- | --- | --- | --- | --- |
| **People with a problem like the person’s (i.e., the social media discloser) could snap out of it if they wanted** |  |  |  |  |  |
| **A problem like the person’s is a sign of personal weakness** |  |  |  |  |  |
| **The person’s problem is not a real medical illness** |  |  |  |  |  |
| **People with a problem like the person’s are dangerous** |  |  |  |  |  |
| **It is best to avoid people with a problem like the person’s so that you do not develop this problem** |  |  |  |  |  |
| **People with a problem like the person’s are unpredictable** |  |  |  |  |  |
| **If I had a problem like the person’s I would not tell anyone** |  |  |  |  |  |
| **I would not employ someone if I knew they had a problem like the person’s** |  |  |  |  |  |
| **I would not vote for a politician if I knew they had suffered a problem like the person’s** |  |  |  |  |  |

| Page Break |  |
| --- | --- |

**Compared to the time before you saw the disclosure, [you are now more or less willing to]**

|  | much less willing | less willing | no change | more willing | much more willing |
| --- | --- | --- | --- | --- | --- |
| **Move next door to people with a problem like the person's (i.e., the social media discloser)** |  |  |  |  |  |
| **Spend an evening socialising with them** |  |  |  |  |  |
| **Make friends with them** |  |  |  |  |  |
| **Work closely with them on a job** |  |  |  |  |  |
| **Have them marry into your family** |  |  |  |  |  |

End of Block: Anti-stigma impact perceptions

# End of Survey

**We thank you for your time spent taking this survey. Your response has been recorded.

If you ever become distressed while completing the survey, you can access confidential support by contacting:**

**UK:**Samaritans helpline on 116 123 (24 hours)
**USA:**National Suicide Prevention Lifeline on 1800 273 8255 (24 hours)
**Canada**: The Canadian Suicide Prevention Service on 1.833.456.4566 (24 hours)
**Australia**: Lifeline on 13 11 14 (24 hours) or Beyond Blue on 1300 22 4636 (24 hours)

**Or, you can contact our research team at zhongjiez@student.unimelb.edu.au. We wish you all the best ❤️**
