# Supplementary material for: Public disclosures of mental health problems on social media and audiences’ self-reported anti-stigma effects
Source: Health Promot Int. 2025 Jan 21;40(1):daae204. doi: 10.1093/heapro/daae204 (PMC11747871; doi:10.1093/heapro/daae204)
Supplement: daae204_suppl_Supplementary_Tables_S1 [file daae204_suppl_supplementary_tables_s1.docx]

# **Supplementary materials:** Table S1 Relationship scores across three groups of disclosers (n=803)

| Characteristic | Celebrity | Influencer | Person | *P* ^a^ |
| --- | --- | --- | --- | --- |
|  | n = 176 | n = 193 | n = 434 |  |
| Empathy, M (SD) | 5.31 (1.13) | 5.19 (1.24) | 5.38 (1.17) | 0.19 |
| Similarity, M (SD) | 3.38 (1.64) | 3.56 (1.75) | 3.84 (1.74) | 0.01 ^b^ |
| Identification, M (SD) | 3.63 (1.63) | 3.73 (1.75) | 4.09 (1.71) | < 0.01 ^c^ |

a. Analyses of Variance (ANOVAs) were employed

b. Post hoc tests (Tukey's Honestly Significant Difference) indicated a significant difference in similarity scores between celebrity and person (*p* < 0.01)

c. Post hoc tests (Tukey's Honestly Significant Difference) indicated a significant difference in identification scores between celebrity and person (*p* < 0.01), and between influencer and person (*p* = 0.03)
