# Supplementary material for: Public disclosures of mental health problems on social media and audiences’ self-reported anti-stigma effects
Source: Health Promot Int. 2025 Jan 21;40(1):daae204. doi: 10.1093/heapro/daae204 (PMC11747871; doi:10.1093/heapro/daae204)
Supplement: daae204_suppl_Supplementary_File2 [file daae204_suppl_supplementary_file2.docx]

# **Supplementary materials:** Summaries of content analysis

| **Top level themes** | **Sub-themes** | **Explanations** | **Example responses** | **Frequency count (N=540), n (%)** |
| --- | --- | --- | --- | --- |
| **None** | Unclear responses |  | *I was very moved by the openness of the disclosure* | 31 (5.74) |
|  | Irrelevant responses |  | *I was sympathizing with her, although I have never been in that situation* | 12 (2.22) |
| **Disclosure of any mental health problem(s)** | Disclosure of an unspecified mental health problem |  | *They disclosed their experience with their mental problem* | 56 (10.37) |
|  | Disclosure of unspecified mental health problems (>one problem |  | *How they are taking medication to deal with their mental health issues and the impact.* | 38 (7.04) |
|  | Disclosure of depression |  | *She explained that her depression was caused by her divorce and abuse from the husband* | 104 (19.26) |
|  | Disclosure of anxiety |  | *it was just them explaining that they are suffering with anxiety but are trying to get through it but may be quiet for a while as a result* | 36 (6.67) |
|  | Disclosure of eating disorder(s) |  | *She shared her experience with anorexia and how she almost died but now she was being taken care by experts* | 13 (2.41) |
|  | Disclosure of bipolar disorder |  | *She described her expiriences in dealing with bipoler disorder and it was affecting her career* | 9 (1.67) |
|  | Disclosure of other problem(s) | Not covered by problems mentioned above | *She posted lots of instagram posts and disclosed about her PTSD diagnosis after giving birth* | 48 (8.89) |
|  | Disclosure of any relapse |  | *she told us her story, when and how it started, why she was afraid to eat and throw up later, how she got better for a while but the disorder came back stronger* | 2 (0.37) |
|  | Disclosure of disclosing decision making process |  | *He felt the need to reveal the problem in order to take time off from work and seek treatment* | 2 (0.37) |
| **Disclosure of suicide related content** | Disclosure of suicide ideation |  | *The person mentioned suicidal ideation* | 20 (3.70) |
|  | Disclosure of suicide attempt |  | *She talked about how he felt every day, what his suicide attempt was like.* | 14 (2.59) |
|  | Disclosure of self harm |  | *She into much greater detail about self harm and how it affected her* | 4 (0.74) |
| **Disclosure of personal experiences, feelings, symptoms** | Disclosure of personal daily experiences | More of an overall description of what the daily life of people with mental health problems looks like | *She often gives glimpses into her daily life and how her anxiety affects her, es well as self compassion and work she's putting in.* | 30 (5.56) |
|  | Disclosure of negative experiences |  | *He mentioned that he is going through a tough time* | 77 (14.26) |
|  | Disclosure of personal feelings | More of an overall description of what people with mental health problems feel about; without any specific mention of positive or negative feelings | *The person showed how they felt and how they are trying to deal with it* | 17 (3.15) |
|  | Disclosure of negative feelings |  | *usually frustration due to mental health related life problems* | 25 (4.63) |
|  | Disclosure of loneliness |  | *This person was talking about how they have been feeling depressed and lonely.* | 9 (1.67) |
|  | Disclosure of any symptom(s) of having the problem(s) |  | *Starting with his own diagnosis, he shared symptoms of his anxiety, and gave some advice to people* | 40 (7.41) |
|  | Disclosure of any reaction(s) from others |  | *mostly how he feel, what he thinks, and how other people think about him* | 4 (0.74) |
| **Disclosure of possible consequence(s) of having the mental health problem(s)** | Disclosure of unspecified consequence(s) | More of an overall description of any consequence(s) the mental health problem(s) might have had on the person; without any specific mention of positive or negative consequences | *The message discussed the diagnosis and what was the cauuse of it and how it has impacted their life* | 31 (5.74) |
|  | Disclosure of any negative consequence(s) |  | *im not really sure as i vaguely remember but it was like i lost a baby due to mental health potentially* | 54 (10.00) |
|  | Getting away from social media as a possible consequence | Explaining their absence from social media due to their problem(s); or informing people of their future absence from social media due to the problem(s) | *It was an almost 10 minute long Tiktok where he talked about his depression and self esteem issues and explained why he took a month long break from the app* | 13 (2.41) |
| **Disclosure of the time duration or trajectory of the mental health problem(s)** | None |  | *He has some psychological problems whole live, because he is morbidly fat and he has to deal with it* | 35 (6.48) |
| **Disclosure of possible reason(s) behind the mental health problem(s)** | Disclosure of any unspecified reason(s) | Mentions of the possible reasons or causes but not into detail | *Types of anxiety and causes.* | 9 (1.67) |
|  | Disclosure of social triggers | Mentions of negative life events that potentially caused the problems; or mentions of broader societal causes/structures | *She discussed how being a celebrity affected her and her mental health.* | 99 (18.33) |
|  | Disclosure of individual level triggers | Mentions of personal psychological traits, or behavioural routines, physical condition(s), or any family history as the cause to the mental health problem(s) | *Not really, it was about having a health problem and developing mental health problems because of it I believe* | 11 (2.04) |
| **Disclosure of coping** | Disclosure of coping - unspecified | Mentions of coping but not into detail | *The person was sharing a lot of information about hey they felt and the steps they took to dealing with depressions and their suicidal thoughts.* | 53 (9.81) |
|  | Disclosure of negative coping |  | *It seemed to be a combination of how things got on top of him, he got depressed, started drinking and abusing drugs.* | 5 (0.93) |
| **Disclosure of help seeking** | Disclosure of receiving help - unspecified | Mentions of receiving help but not into detail as to what sort of help | *He told followers about his struggles with depression and why he had been quiet on social media as he was battling and seeking help* | 8 (1.48) |
|  | Disclosure of receiving informal help | Mentions of receiving help from social networks such as friends, family or social media followers | *He gave a brief run down of when it started and how his family stood by him and all.* | 7 (1.30) |
|  | Disclosure of receiving professional help - unspecified | Mentions of receiving professional help but not into detail | *The YouTuber had suffered from depression and received medical care to overcome the problem.* | 22 (4.07) |
|  | Disclosure of receiving psychotherapy |  | *They just tweeted that they had been struggling with their mental health for a while and adviced others to see a therapist like they did* | 21 (3.89) |
|  | Disclosure of taking medication |  | *This influencer is always talking about how her daily life was affected but how ok she feels right now with her medication.* | 27 (5.00) |
|  | Disclosure of receiving institutional care/hospitalisation |  | *The person suffered depression such that they got admitted with treatment to help maintain themselves and stabilize* | 12 (2.22) |
|  | Disclosure of seeing psychiatrists |  | *He thanked his fans for their support and told them he was going to take a break. He also mentioned it was his first time being diagnosed by a doctor.* | 5 (0.93) |
|  | Desire for any help |  | *Asking for help* | 8 (1.48) |
| **Disclosure of any stigma experiences** | Disclosure of self stigma experiences |  | *She talked about postpartum depression and how she didn't feel comfortable admitting she had it* | 12 (2.22) |
|  | Disclosure of being stigmatised by others |  | *She used previous pictures of how her body used to depress her and how people would always make bad remarks onher body* | 5 (0.93) |
| **Disclosure of recovery** | Disclosure of recovery - unspecified | Mentions of recovery but not into detail | *I cannot remember much word for word but she basically spoke about what she has been experiencing as a person readjusting and living with an eating disorder. How it has affected her and those around her.* | 62 (11.48) |
|  | Disclosure of less self stigma over time | Self stigma reduced through self acceptance | *She initially disclosed that she acknowledges her problem and that she has been getting better over time after accepting it.* | 5 (0.93) |
|  | Social media as a tool for recovery |  | *The person was documenting their journey of coping with binge eating and posting was an accountability measure.* | 2 (0.37) |
| **Encouraging messages** | Encouraging - unspecified | Providing encouragement, mental support to people who might have the same problem(s) | *it involved encouraging message, the issues the person was experiencing and what steps they are taking to deal with it* | 2 (0.37) |
|  | Encouraging help seeking - professional help |  | *Encouraging to get professional help, trying to make the subject more normal* | 20 (3.70) |
|  | Encouraging help seeking - informal help |  | *My friend made a post on Instagram about a person saying a quote regarding on mental health and how to cope and understand that mental health can be recover with friends and family.* | 5 (0.93) |
|  | Encouraging help seeking - general unspecified help |  | *People should get help* | 24 (4.44) |
|  | Encouraging opening up | Encouraging people to speak up their problem(s) | *The message was to try help others to talk about their mental health and not be ashamed.* | 5 (0.93) |
|  | Encouraging solidarity | Telling that you are not alone | *He spoke about realizing he was not alone in having depression, and that other people would deserve to kknow it as well* | 11 (2.04) |
| **Altruism messages** | Sharing advice on positive/effective coping strategies |  | *advice on where to get help was offered* | 18 (3.33) |
|  | Willingness to offer help |  | *THEY WERE REACHING OUT TO HELP PEOPLS* | 2 (0.37) |
| **Advocacy messages** | Advocacy for people with mental health problem(s) |  | *It was more of an asking to be kind towards others who deal with mental health issues* | 5 (0.93) |
|  | Anti-stigma advocacy | Including different manifestations of stigmatisation such as laziness or difference perceptions | *Something like,i am good person,you don't need to be afraid because i am different.* | 17 (3.15) |
|  | Raising mental health related awarenesses |  | *He was trying to bring awareness to his condition and* | 22 (4.07) |
|  | Providing mental health related educational information | Offering mental health related fact data, or giving advice on how to recognise and support someone going through mental health problem(s) | *educates people on how they should behave towards mentally ill people* | 20 (3.70) |
|  | Normalising mental health related issues | Normalising mental health problem(s), or help-seeking behavior | *Reassuring that mental health issues are normal and encouraging others to talk openly about them* | 14 (2.59) |
| **Impact of social media on mental health** | None |  | *It might have been covered by one of the earlier options, but they also spoke of the impact of social media on their mental health* | 1 (0.19) |
| **Life reflections** | None |  | *Overcoming depression and finding sense/motivation in life* | 8 (1.48) |
| **Others** | Questioning normality | Stating distinctions between normality and abnormality from the perspective of lived experience | *The message stated 'normal' people don't know what it's like to live with depression* | 1 (0.19) |
|  | Anti-social messages | Defiant messages towards society | *defiant message towards society* | 1 (0.19) |
|  | Disclosure on social media as a way to beg |  | *Every once in a while he claims to suffer from depression. They even made a stupid statement dance mix about it. I really think it's just a begging implement.* | 1 (0.19) |
